# Inter-Examiner Disagreement for Assessing Cervical Multifidus Ultrasound Metrics Is Associated with Body Composition Features

**DOI:** 10.3390/s23031213

**Published:** 2023-01-20

**Authors:** Umut Varol, Marcos José Navarro-Santana, Sonia Gómez-Sánchez, Gustavo Plaza-Manzano, Elena Sánchez-Jiménez, Juan Antonio Valera-Calero

**Affiliations:** 1 Escuela Internacional de Doctorado, Universidad Rey Juan Carlos, 29222 Alcorcón, Spain; 2 Faculty of Health, Universidad Católica de Ávila, C/Canteros, s/n, 05005 Ávila, Spain; 3 Department of Radiology, Rehabilitation and Physiotherapy, Universidad Complutense de Madrid, 28040 Madrid, Spain; 4 Grupo InPhysio, Instituto de Investigación Sanitaria del Hospital Clínico San Carlos (IdISSC), 28040 Madrid, Spain

**Keywords:** body composition, fat, lean mass, ultrasound imaging, spine, diagnostic accuracy studies, reliability

## Abstract

Ultrasound imaging (US) is a biosensing technique that is widely used in several healthcare disciplines (including physiotherapy) for assessing multiple muscle metrics, such as muscle morphology and quality. Since all biosensors need to be tested in order to demonstrate their reliability, accuracy, sensitivity, and specificity, identifying factors that affect their diagnostic accuracy is essential. Since previous studies analyzed the impact of sociodemographic but not body composition characteristics in US errors, this study aimed to assess whether body composition metrics are associated with ultrasound measurement errors. B-mode images of the lumbar multifidus muscle at the L5 level were acquired and analyzed in 47 healthy volunteers by two examiners (one experienced and one novice). The cross-sectional area, muscle perimeter, and mean echo intensity were calculated bilaterally. A correlation analysis and a multivariate linear regression model were used for assessing the inter-examiner differences with respect to body composition metrics. The results demonstrated good-to-excellent reliability estimates for the cross-sectional area, muscle perimeter, aspect ratio, roundness, circularity, and mean brightness metrics (all ICC > 0.85). However, solidity showed unacceptable reliability (ICC < 0.7). Age, height, total lean mass, trunk lean mass, and water volume were associated with inter-examiner disagreement on mean echo intensity. Cross-sectional area, perimeter, and roundness measurement errors were associated with lean mass and water volume.

## 1. Introduction

The anatomy, function, and clinical relevance of the posterior deep neck muscles have been widely described in the literature [1,2,3,4,5,6,7,8,9,10,11]. The cervical multifidus muscles are divided into superficial and deep fascicles, originating from the facet capsule of C4–C7 and inserted on the spinous process of C2 and the laminae of the superior vertebrae (just anterior to the base of the spinous process), respectively [1]. In addition to the small moment arms in extension, rotation, and lateral flexion movements [1,2], these muscles play a relevant role in the segmental stabilization of the spine [2].

From a clinical perspective, several studies have analyzed the correlation between the morphology and histology of the cervical multifidus and investigated differences between clinical populations and healthy or asymptomatic subjects [2,3,4,5,6,7,8,9,10,11]. For instance, the intramuscular fatty infiltration percentage seems to be associated with cervical myelopathy (i.e., impaired sensorimotor function) [3], cervical spondylotic radiculopathy (i.e., poorer postural stability) [4], and non-specific chronic neck pain (i.e., greater disability) [5] severity. In addition to the intramuscular fatty infiltration, muscle morphology [6,7,8] and muscle function [9,10,11] are also relevant metrics to consider in several musculoskeletal conditions involving neck pain. Thus, this muscle is targeted in therapeutic interventions such as dry needling [12,13] and motor control exercises [14,15] for reducing neck pain symptoms, supporting the need for its correct identification with US to correctly guide the needle and to accurately measure muscle changes during and after motor control exercises, respectively.

Although most of the studies analyzing the size, shape, and histology of the cervical multifidus muscle have been conducted using magnetic resonance imaging (MRI) [16,17,18,19,20,21], there is an increasing tendency to use ultrasound (US) imaging examinations to quantify the morphological (e.g., thickness, cross-sectional area, perimeter, shape descriptors, volume) and histological (e.g., quantification of intramuscular fatty infiltration and mean echo intensity as muscle quality indicators) characteristics of this muscle [22,23,24,25,26,27,28,29,30].

One likely explanation for this tendency is the advantages of US over other imaging methods. In comparison with MRI, computed tomography (CT), or X-ray imaging, US is faster, safer (as no ionizing radiation is involved in the imaging acquisition), more cost-effective, provides real-time information, and is generally more accessible [31]. In addition to these advantages, clinicians currently demand biosensors with acceptable utility (i.e., reliability, specificity, sensitivity, and validity) as a complement or alternative to manual explorations [31,32]. Since recent developments in US technology allow the measurement of multiple objective metrics by using Doppler (for detecting and analyzing flows) [33], M-mode (for real-time feedback during muscle contractions or for measuring thickness changes) [34], or elastography (for measuring stiffness metrics) [35], along with the assessment of large structures that previously could not be entirely visualized in a single image (i.e., panoramic US) [36], this tool is one of the most attractive alternatives to these imaging methods to meet these demands. 

However, US is not free of disadvantages. Some of the main limitations of this tool are related to the US physics principles limiting the visualization of several locations where sound cannot be reflected [37], the operator-dependence (which is closely associated with the examiner’s experience and the region of interest’s location in the image) [38], and the histological properties of the tissues [28]. Regarding the latter issue, US reliability estimates are worse in clinical populations compared with healthy subjects following the same procedures [25,26,27,28,39]. One potential reason proposed in previous research is related to the increased intramuscular fatty infiltration that occurs in patients with whiplash-associated disorders and causes difficulty in delimiting the muscle contours [28,39]. Additionally, another study seeking sociodemographic and muscular factors associated with inter-examiner errors found that age was a highly relevant factor [29]. 

There are three main reasons supporting the rationale for conducting this study: First, in the only previous study assessing the correlation of US errors with sociodemographic, clinical, muscular, and histological factors, the authors found no significant associations with clinical severity. Secondly, only body mass index was assessed as a body composition indicator. Finally, previous research suggests that body composition changes associated with aging [40,41,42,43,44,45] could be the real reason behind the visualization difficulties. Considering this background, this study aimed to identify which body composition metrics (analyzed with a valid instrument) contribute to the variance in inter-examiner disagreement for measuring cervical multifidus US characteristics in a sample of healthy volunteers.

## 2. Materials and Methods

### 2.1. Study Design

This was a cross-sectional observational study designed for assessing the diagnostic accuracy of US. This study design was conceived to quantify the contributions of sociodemographic factors (i.e., age and sex) and body composition (i.e., height, weight, body mass index, percentage of total corporal fat, percentage of total corporal lean mass, total water mass, and body impedance) to the inter-examiner disagreement with respect to US cervical multifidus muscle measurements (i.e., muscle morphology: cross-sectional area, perimeter, and shape descriptors; muscle quality: mean echo intensity, and intramuscular muscle fat infiltration). This report followed the Standards for the Reporting of Diagnostic Accuracy Studies (STARD) guidelines and checklist [46].

### 2.2. Participants

The sample was recruited from a private university located in Ávila (Spain). Between August 2022 and September 2022, local announcements were posted around the campus explaining the aims and procedures of this research. The data collection started in October 2022 and finished in November 2022. The eligibility criteria only required no history of neck pain during the previous year and a minimum age limit of 18 years old. No height, weight, or upper age limit criteria were established, so as to recruit the most heterogeneous sample possible to ensure the results’ generalizability.

Exclusion criteria were the use of any pharmacological treatment affecting muscle tone, history of neck or spine surgery, cervical radiculopathy or myelopathy, confirmation of severe degenerative cervical radiological findings, or history of any musculoskeletal or medical condition (either local—e.g., whiplash-associated disorders, spondylolysis, spondylolisthesis, tumors, or fractures—or widespread, e.g., fibromyalgia). Once the eligibility criteria were verified by one independent researcher, participants had to read and sign an informed written consent waiver to be included in the data collection.

### 2.3. Sample Size Estimation

The minimum sample size calculation was conducted following the directives proposed by Green [47] for regression models, which has demonstrated acceptable validity and power for detecting associations and factor analyses [48]. According to the formula 50 + 8 *m* (where *m* is the number of independent variables introduced in the regression analysis, and introducing no more than 5 predictors to avoid accuracy overestimation bias [47,48]), a minimum of 90 data points could be considered appropriate. Therefore, the minimum number of participants for this study was set at 45, since both sides were measured and no significant side-to-side asymmetries were expected in healthy participants based on previous studies [25,26,27].

### 2.4. Data Collection

#### 2.4.1. Sociodemographic and Body Composition Data

The InBody 270 device (Biospace Inc., Los Angeles, CA, USA) was used for assessing the participants’ body composition. This device consists of a multi-frequency system based on bioimpedance technology, allowing the measurement of total water volume (L), weight (kg), body fat (kg and %), body mass index (BMI = $\frac{\mathrm{weight}\left(\mathrm{kg}\right)}{\mathrm{height}\;{\left(m\right)}^{2}\;}$), and muscle fat (kg and %). We also introduced the participants’ height without footwear (m), age (years), and sex (male/female) and exported all of the results to a standardized document.

This device was used because a previous study reported almost perfect association with a gold-standard method (dual-energy X-ray absorptiometry, r > 0.97) and excellent reliability (ICC > 0.98) [49]. All measurements were carried out by an independent investigator between 9:00 and 11:00 a.m., weighing all participants in light clothing according to the protocol described by the authors of the validity study [49].

#### 2.4.2. Examiners

All US measurements were performed by two examiners—one experienced (>10 years of experience using musculoskeletal US) and one novice (>10 years of clinical experience, but no previous US experience). The rationale for assessing only inter-examiner reliability was based on the almost perfect intra-examiner reliability estimates reported previously with small errors for image acquisition [25]. Additionally, we selected one experienced examiner as a reference and one novel examiner, as this combination showed the lowest reliability estimates and greatest errors for imaging measurement [25].

As conducted in previous studies, the experienced examiner trained the novice on basic US concepts and the specific procedures for this study (patients’ positioning, transducer placement, US settings, muscle contouring, etc.) in a 10 h training program distributed between two sessions [25,26,27,28,50]. Before starting the study, the experienced examiner ensured that the novice acquired the skills needed to perform the measurements by conducting the full protocol satisfactorily.

#### 2.4.3. Ultrasound Imaging Acquisition

All ultrasound images were acquired with a Logiq P9 device and an ML-6-15-D linear 6–15 MHz transducer (General Electric Healthcare, Milwaukee, WI, USA). The console settings were also standard for all of the imaging acquisitions (frequency = 12 MHz, gain = 65 dB, and depth = 4.5 cm).

Immediately after conducting the body composition analyses, participants were placed in the prone position with a pillow under their feet and their arms resting with 90° shoulder abduction and 90° elbow flexion. The head and neck were stabilized using the plinth’s facial hole in a craniocervical flexion position to reduce the cervical lordosis. 

The imaging acquisition procedure was conducted as described in previous studies [25,26,27,28,29], selecting the C4–C5 level and applying the smallest possible pressure with the transducer. 

This procedure was conducted for both the left and the right sides once by each examiner, in a randomized order (for side and examiner), allowing only the examiner acquiring the images to be present in the room in order to ensure the blinding.

#### 2.4.4. Measurement of Muscle Morphology and Quality

An independent researcher codified, saved and—after exporting the acquired images to DICOM format—sent the files to both examiners. Each examiner measured the images that they acquired in a randomized order. To ensure the blinding, no information was shared between the examiners during this process.

**Figure 1:**
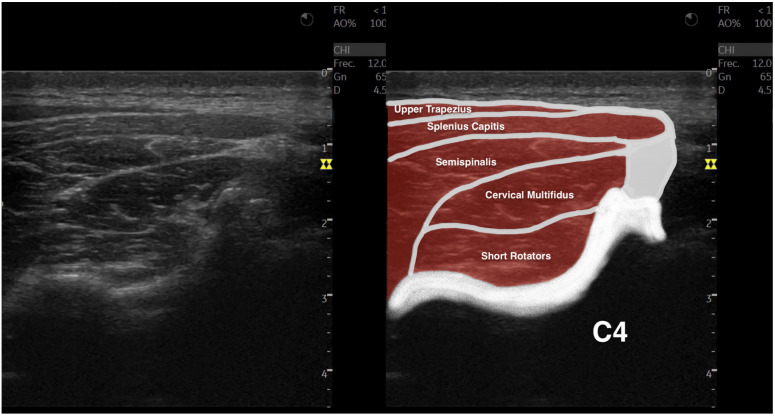
Ultrasound imaging acquisition and measurement: Example image illustrating the cervical multifidus muscle acquisition at the C4–C5 level (left), and with muscular structures labeled (right).

All images were analyzed using the ImageJ offline DICOM software (National Institutes of Health, Bethesda, MD, USA, v.1.53a). After transforming the images to 32-bit images (which is a 256 grayscale image), the cervical multifidus was contoured, avoiding the inclusion of bone or surrounding fascia, as shown in Figure 1, within the limits described in the literature (i.e., internal fascia between the cervical multifidus muscle with short rotators and the semispinalis muscles and the spinous process) [25,26,27,28]. Finally, the muscle morphology (cross-sectional area in mm^2^ and perimeter in mm), shape (circularity was calculated as 4π × area/perimeter^2^—values range from 0 to 1, where a value of 1 indicates a perfect circle; aspect ratio was calculated as the division between the major axis and the minor axis, and roundness was calculated as 4 × area/(π × major axis^2^)), and muscle quality (mean echo intensity, calculated as the mean average brightness in this 256 grayscale image within the region of interest contoured) metrics were calculated [25,26,27,28].

### 2.5. Statistical Analysis 

All analyses were conducted using the Statistical Package for the Social Sciences (SPSS v.27, Armonk, NY, USA) for Mac OS, setting the significance level at *p* < 0.05 for all of the analyses. Firstly, the data distribution was verified using histograms and Shapiro–Wilk tests for continuous variables; *p*-values < 0.05 were considered to be non-normally distributed, while *p* > 0.05 indicated normal distribution [51]. 

Secondly, descriptive statistics were used for reporting the total sample’s characteristics. Categorical data were reported as the frequency and percentage for each category (e.g., number and percentage of women and men). Continuous variables were reported using central tendency metrics (i.e., mean for normal variables and median for non-normal variables) and dispersion metrics (i.e., standard deviation for normal variables and interquartile range for non-normal variables). Additionally, gender and side differences (if applicable) were analyzed using student’s *t*-test for independent samples, reporting the mean difference with a 95% confidence interval and considering a *p* value < 0.05 as statistically significant. 

The inter-examiner reliability analysis consisted of (1) central tendency and dispersion for each metric obtained by each examiner, (2) absolute error between examiners (absolute error was calculated because signs could underestimate the disagreement magnitude), (3) intraclass correlation coefficients (ICC_3,2_ calculated with a 2-way mixed model, consistency type), (4) standard error of measurements (SEM= $\sqrt{1}-\mathrm{ICC}$ × standard deviation of mean scores), and (5) minimum detectable changes (MDC_95_ = SEM × 1.96 × $\sqrt{2}$) [52].

For assessing the associations of the sociodemographic and body composition characteristics with US errors, a Pearson’s correlation matrix was calculated. Pearson’s correlation coefficients (r) were used to analyze the direction and strength of these associations and to identify multicollinearity and shared variance between variables (if r > 0.80) [53,54].

## 3. Results

Of the potential 50 participants initially interested in participating, 3 were excluded due to history of clinically relevant neck pain episodes within the previous year (*n* = 3). Since 47 asymptomatic volunteers were finally included in the data collection, and both the left and right sides were analyzed, 94 cervical multifidus muscles were analyzed.

Table 1 describes the body composition and cervical multifidus characteristics of the participants. Although males and females had comparable body mass index (*p* > 0.05), males were older (*p* < 0.05), taller (*p* < 0.001), heavier (*p* < 0.001), and showed greater water volume (*p* < 0.001), fat mass (trunk, *p* < 0.01), and lean mass (trunk and total, *p* < 0.001). Regarding the cervical multifidus muscle, results showed significant gender differences for muscle size (cross sectional area *p* < 0.001 and perimeter *p* < 0.001), circularity (*p* < 0.001), and mean brightness (*p* < 0.05). No side-to-side differences were observed in the sample (all metrics, *p* > 0.05).

Table 2 summarizes the statistical estimates of the inter-examiner reliability data. The results showed excellent ICC estimates for measuring muscle cross-sectional area and mean brightness (ICC = 0.955 and ICC = 0.983, respectively), and good estimates for measuring muscle perimeter (ICC = 0.893) and most of the shape descriptors (ICC = 0.856–0.884). However, solidity did not reach the minimal acceptable reliability (ICC < 0.7). Indicative MDC values are also detailed to show whether changes in future research with longitudinal designs assessing the effects of specific interventions on these metrics would be attributable to real changes (if changes are greater than MDCs) or measurement errors (if changes are smaller than MDCs).

Table 3 describes the associations between body composition characteristics and inter-examiner errors for each US metric. Weight, body mass index, and fat mass (either total or trunk) were not associated with US measurement errors (all *p* > 0.05). However, several body composition features were significantly associated with errors of some US metrics. For instance, mean brightness error showed the highest number of body composition factors associated with inter-examiner disagreement, including older age (*p* < 0.01), taller subjects (*p* < 0.05), greater lean mass (total and trunk, *p* < 0.01), and greater water volume (*p* < 0.05). Additionally, the results demonstrated lean mass to be positively associated with cross-sectional area (total *p* < 0.01 and trunk *p* < 0.05), perimeter and roundness errors (both *p* < 0.05), and water volume with cross-sectional area errors (*p* < 0.05). Additionally, multiple internal associations were found among the metrics’ errors (*p* < 0.05).

## 4. Discussion

To the best of the authors’ knowledge, this is the first study quantifying the contribution of body composition metrics to US errors. In general, we found several gender differences for cervical multifidus size, shape, and brightness, but no side-to-side asymmetries. Reliability data showed good-to-excellent statistical estimates for inter-examiner reliability, but solidity estimations did not reach the minimum ICC score to be considered acceptably reliable. Additionally, mean echo intensity reliability was the most dependent metric on the participants’ body composition characteristics, since up to five factors were associated with brightness errors (i.e., age, height, total lean mass, trunk lean mass, and water volume). Cross-sectional area, perimeter, and roundness measurement errors were also associated with lean mass and water volume. 

The rationale for conducting this study was based on the importance of US physics principles for the correct interpretation of the images and better understanding of imaging limitations [55,56,57,58]. The physical mechanism of this imaging tool is based on non-ionizing mechanical and longitudinal sound waves produced by the vibration of piezoelectric materials during electric current exposure at a rate of 2–18 MHz in most US devices [59].

These sound waves penetrate from the transducer to the tissues and use the sound reflection to build a two-dimensional 256 grayscale image, with 0 being a black pixel and 255 a white pixel. This brightness depends on the reflection strength and several other factors. First, not all of the sound emitted from the transducer is reflected, as part of the US is lost due to the interaction with the tissues (generating a thermic response derived from the vibration, which is the main principle of therapeutic US), and part is lost due to refraction (i.e., changes in the US waves’ direction during certain interactions with the tissues, depending on their shape and histological characteristics) [55]. For these reasons, US waves are attenuated at deeper structures and, therefore, US reflection decreases proportionally. 

Although depth is one of the most important limitations of US, operators can change and modulate the US pulses emitted from the transducer and the console settings for better visualization [60,61,62]. Since higher frequencies interact more with the tissues (resulting in greater attenuation in deeper structures), reducing the frequency improves the visualization of deep structures. In fact, curvilinear transducers (which provide a trapezoidal image, expanding the field of view proportionally to the structures’ depth—in contrast with linear transducers, which provide a rectangular image with constant width) emit the lowest range of US frequencies to facilitate the visualization of deep structures [63]. However, the inconvenience of using lower frequencies is that, since fewer interactions are produced, the image quality is generally poorer [56].

Moreover, dynamic range (defined as the ratio between the highest and the lowest brightness), gain (i.e., pixels’ displacement to whiter or darker brightness), and focus (i.e., the depth range where better image quality is needed) are set in every exam to improve the image quality [60,61,62].

In addition to the US amplitude, reflection also depends on the tissues’ acoustic impedances (a property associated with the density and propagation speed of the sound) [56]. Tissues with large acoustic impedance differences result in stronger reflection (often reflecting the totality of the US with no further sound penetration) and, therefore, generate white pixels (with posterior acoustic shadows if all of the sound is reflected). In contrast, if no acoustic impedance differences occur, there is no US reflection, and the resulting image is black [56]. Since most of the soft tissues assessed in musculoskeletal US imaging have small acoustic impedance differences, these structures show small brightness differences in the grayscale image. For this reason, histological changes may play a relevant role in image compounding. 

A previous study demonstrated that age was the most significant factor explaining the inter-examiner disagreement during US measurements of cross-sectional area, mean echo intensity, and fatty infiltration estimation in deep neck muscles [29]. However, despite aging being a factor that is clearly associated with changes in body composition, such as body fat gain and muscle loss [55], no previous studies have analyzed the association between easily measurable body composition metrics and US errors. In fact, considering that a previous study [56] reported that aging was associated with slow weight loss that becomes faster after the age of 75, an annual fat mass gain of 0.40% after the age of ~45, and a significant lean mass loss (especially in men and in leg muscles) in the general population, analyzing these potential associations may improve the efficiency of US resources by avoiding their use in unreliable conditions. 

Although we did not find age to be associated with inter-examiner disagreement for most of the US metrics (maybe because of the limited age range of our sample), we found some body composition metrics to be associated with measurement errors. As expected based on the rationale provided previously, we found the mean brightness disagreement to be associated with age, height, total lean mass, trunk lean mass, and water volume. One possible reason for the interaction of these five features might be the significant internal associations between these metrics. 

In addition to body composition changes associated with aging, other factors may influence the reliability of US. For instance, previous reliability and validity studies assessing deep paraspinal muscles found poorer statistical estimates following the same procedures in clinical populations compared with healthy and asymptomatic subjects [7,8,25,26,27,28,29]. Although technological advances have significantly improved the US image quality and diagnostic accuracy [29], other factors associated with the histological characteristics of the tissues (especially in chronic pain syndromes involving fiber-type changes [64,65], muscle mass loss [66,67,68], and greater intramuscular fatty infiltration [69,70]) and the operator ability (e.g., operator-applied compression with the transducer [71]) could also potentially be determining factors influencing the contouring of the region of interest [39]. 

### Limitations

Several limitations should be acknowledged in this study. First, future studies should include larger sample sizes and increase the diversity of both body composition and sociodemographic factors to corroborate these findings. Secondly, we limited our recruitment strategy to healthy volunteers without chronic pain conditions. Although this strategy was useful to isolate body composition and sociodemographic factors associated with measurement errors, further research is needed to quantify the contribution of clinical severity (in terms of disability, pain intensity, duration of symptoms, pain extent, etc.) to measurement errors. Thus, only two examiners and a single US device were involved in this study. Further research is needed for analyzing whether other factors related to the examiners (e.g., time of exam, pressure with the transducer over the skin, and years of experience), number of records, environmental conditions, and US settings and devices contribute to the measurement errors. Finally, we only assessed the cervical multifidus muscle at a specific level. In future studies, other levels and musculoskeletal structures should be tested.

## 5. Conclusions

This study assessing a sample of healthy volunteers found no side-to-side asymmetries, but there were significant gender differences in the cervical multifidus muscle’s size, shape, and brightness at the C4–C5 level. Good-to-excellent inter-examiner reliability estimates were found for the cross-sectional area, muscle perimeter, aspect ratio, roundness, circularity, and mean brightness metrics. However, solidity showed unacceptable reliability. The multivariate correlation matrix showed age, height, total lean mass, trunk lean mass, and water volume to be associated with inter-examiner disagreement with respect to mean echo intensity. Cross sectional area, perimeter, and roundness measurement errors were associated with lean mass and water volume.

## Figures and Tables

**Table 1:** Participants’ body composition and US characteristics.

Variables	Total Sample (*n* = 47)	Gender		Side	
		Male (*n* = 22)	Female (*n* = 25)	Left (*n* = 47)	Right (*n* = 47)
Body Composition Characteristics					
Age (y) *	21.0 ± 5.7	23.3 ± 7.5	20.1 ± 2.3	-	-
Height (m) **	1.72 ± 0.08	1.77 ± 0.05	1.67 ± 0.06	-	-
Weight (kg) **	72.0 ± 14.0	78.5 ± 10.8	65.8 ± 14.1	-	-
Body Mass Index (kg/m^2^)	24.3 ± 4.5	25.0 ± 3.9	23.7 ± 5.1	-	-
Fat Mass					
Total Mass (kg) *	18.3 ± 9.7	15.6 ± 8.6	20.8 ± 10.2	-	-
Trunk Mass (kg)	9.2 ± 5.2	8.2 ± 4.8	10.3 ± 5.5	-	-
Lean Mass					
Total Mass (kg) **	37.6 ± 15.2	44.5 ± 15.6	30.1 ± 11.6	-	-
Trunk Mass (kg) **	25.8 ± 6.2	30.0 ± 5.3	21.9 ± 4.2	-	-
Water Volume (L) **	39.6 ± 7.6	46.0 ± 4.1	33.6 ± 4.6	-	-
Cervical Multifidus Ultrasound Characteristics					
Cross-Sectional Area (cm^2^) **	1.28 ± 0.35	1.49 ± 0.36	1.09 ± 0.19	1.28 ± 0.35	1.29 ± 0.35
Muscle Perimeter (cm) **	5.0 ± 0.5	5.3 ± 0.4	4.7 ± 0.4	5.0 ± 0.50	5.0 ± 0.52
Circularity (0–1) **	0.54 ± 0.06	0.56 ± 0.06	0.53 ± 0.05	0.54 ± 0.06	0.55 ± 0.05
Aspect Ratio	2.91 ± 0.51	2.85 ± 0.52	2.97 ± 0.50	2.93 ± 0.51	2.89 ± 0.52
Roundness	0.35 ± 0.06	0.36 ± 0.06	0.34 ± 0.06	0.35 ± 0.06	0.35 ± 0.06
Solidity	0.94 ± 0.03	0.94 ± 0.03	0.93 ± 0.03	0.93 ± 0.03	0.94 ± 0.03
Mean Echo Intensity (0–255) *	44.7 ± 14.5	40.8 ± 12.5	48.5 ± 15.3	44.1 ± 14.4	45.4 ± 14.7

* Statistically significant gender differences (*p* < 0.05). ** Statistically significant gender differences (*p* < 0.001).

**Table 2:** Inter-examiner reliability for the anterior scalene US metrics.

Variables	Experienced Examiner	Novel Examiner	Absolute Error	ICC_3,2_ (95% CI)	SEM	MDC_95_
Cross-Sectional Area (cm^2^)	1.29 ± 0.35	1.28 ± 0.35	0.10 ± 0.10	0.955 (0.931; 0.971)	0.07	0.21
Muscle Perimeter (cm)	5.0 ± 0.5	5.0 ± 0.5	0.3 ± 0.2	0.893 (0.833; 0.931)	0.2	0.5
Circularity (0–1)	0.55 ± 0.06	0.54 ± 0.06	0.03 ± 0.03	0.856 (0.777; 0.907)	0.02	0.06
Aspect Ratio	2.92 ± 0.53	2.91 ± 0.57	0.27 ± 0.26	0.863 (0.788; 0.912)	0.20	0.54
Roundness	0.35 ± 0.06	0.35 ± 0.07	0.03 ± 0.03	0.884 (0.820; 0.925)	0.02	0.06
Solidity	0.94 ± 0.03	0.93 ± 0.03	0.03 ± 0.02	0.673 (0.494; 0.789)	0.02	0.05
Mean Echo Intensity (0–255)	44.3 ± 14.6	45.3 ± 14.5	2.5 ± 3.0	0.983 (0.974; 0.989)	1.90	5.3

SEM and MDC_95_ are expressed in the units described for each parameter.

**Table 3:** Pearson’s product–moment correlation matrix.

	1	2	3	4	5	6	7	8	9	10	11	12	13	14	15
1. Age															
2. Height	0.392 **														
3. Weight	0.359 **	0.381 **													
4. Body Mass Index	n.s.	n.s.	0.885 **												
5. Total Fat Mass	n.s.	−0.332 **	0.659 **	0.877 **											
6. Trunk Fat Mass	n.s.	−0.252 *	0.717 **	0.897 **	0.990 **										
7. Total Lean Mass	0.529 **	0.588 **	0.488 **	0.233 *	n.s.	n.s.									
8. Trunk Lean Mass	0.567 **	0.692 **	0.672 **	0.378 **	n.s.	n.s.	0.926 **								
9. Water Volume	0.457 **	0.754 **	0.715 **	0.392 **	n.s.	n.s.	0.663 **	0.882 **							
10. Cross-Sectional Area Error	n.s.	n.s.	n.s.	n.s.	n.s.	n.s.	n.s.	n.s.	n.s.						
11. Muscle Perimeter Error	n.s.	n.s.	n.s.	n.s.	n.s.	n.s.	0.298 **	0.274 *	0.235 *	0.551 **					
12. Circularity Error	n.s.	n.s.	n.s.	n.s.	n.s.	n.s.	0.253 *	n.s.	n.s.	n.s.	0.380 **				
13. Aspect Ratio Error	n.s.	n.s.	n.s.	n.s.	n.s.	n.s.	n.s.	n.s.	n.s.	n.s.	n.s.	0.242 *			
14. Roundness Error	n.s.	n.s.	n.s.	n.s.	n.s.	n.s.	0.220 *	n.s.	n.s.	n.s.	n.s.	0.239 *	0.851 **		
15. Solidity Error	n.s.	n.s.	n.s.	n.s.	n.s.	n.s.	n.s.	n.s.	n.s.	n.s.	0.279 *	0.434 **	n.s.	n.s.	
16. Mean Echo Intensity Error	0.286 **	0.253 *	n.s.	n.s.	n.s.	n.s.	0.432 **	0.378 **	0.238 *	n.s.	n.s.	0.221 *	0.301 **	0.311 **	n.s.

Abbreviations: n.s. = non-significant; * = *p* < 0.05; ** = *p* < 0.01.

## Data Availability

Not applicable.
